# Whole transcriptome analysis of peripheral blood identifies systemic innate immune responses and translation inhibition in subclinical Holstein cattle naturally infected with *Mycobacterium avium* subsp. *paratuberculosis*

**DOI:** 10.3389/fimmu.2026.1744978

**Published:** 2026-05-20

**Authors:** Arrate Prado-López, Gerard Badia-Bringué, Rosana Torremocha, Alejandra I. Navarro León, Rosa Casais, Marta Alonso-Hearn

**Affiliations:** 1 Department of Animal Science, NEIKER-Basque institute for Agricultural research and Development, Basque Research and Tecnology Alliance (BRTA), Derio, Bizkaia, Spain; 2 Doctoral Program in Immunology, Microbiology, and Parasitology, Euskal Herriko Unibertsitatea (EHU), Leioa, Bizkaia, Spain; 3 Genomic Unit, Scientific Park of Madrid, Madrid, Spain; 4 Centre of Animal Biotechnology, Regional Service of Agricultural Research and Development (SERIDA), Deva, Asturias, Spain

**Keywords:** biomarkers, innate immune response, paratuberculosis, RNA-Seq, translation inhibition

## Abstract

Bovine paratuberculosis (PTB), caused by *Mycobacterium avium* subsp. *paratuberculosis* (MAP), is a chronic granulomatous intestinal disease that leads to substantial economic losses in the global dairy industry. Current diagnostic tests have limited sensitivity, as they can reliably detect only animals that have advanced stages of disease characterized by diffuse lesions and with the presence of clinical signs, but fail to identify those in earlier or subclinical stages, with focal or multifocal lesions in gut tissues. Previous studies have suggested that multifocal granulomas prevent lesions progression, but the molecular mechanisms involved in the establishment and maintenance of a chronic MAP infection are not fully understood. This study aimed to compare the whole transcriptomic profiles of peripheral blood (PB) samples from Holstein cattle with multifocal lesions and those without lesions in gut tissues. Total RNA was extracted from samples from PB samples collected from 11 cows with multifocal lesions and no clinical signs of PTB, and from 4 control cows without lesions that tested negative in several PTB diagnostic assays. RNA libraries were prepared using 250 ng of RNA with the Illumina NEBNext Ultra Directional RNA library preparation kit and sequenced on an Illumina NextSeq sequencer. On average, 34.08 million raw reads were sequenced from PB samples. In cows with multifocal lesions compared with control cows, 1,272 differentially expressed genes (DEGs) were identified in PB. Protein-to-protein interaction analysis revealed that several DEGs were highly interconnected and associated with molecular processes related to splicing and translation inhibition, as well as with the activation of a robust innate immune response in PB. Overall, this study provides new insights into MAP pathogenesis and identifies potential biomarkers and therapeutic targets.

## 1 Introduction

Bovine paratuberculosis (PTB), also known as Johne’s disease, is a chronic granulomatous enteritis caused by *Mycobacterium avium* subsp. *paratuberculosis* (MAP) that affects both domestic and wild ruminants worldwide. The main clinical signs in cattle include chronic diarrhea, progressive weight loss, and decreased milk production. PTB occurs worldwide, and estimates show that over 50% of cattle herds in Europe and North America are infected (1). The disease is responsible for substantial economic losses, estimated at approximately US$4 billion annually worldwide (2), including US$364 million in Europe and around US$12 million per year in Spain (3). In addition to production losses, PTB can lead to fertility problems and increased susceptibility to other diseases such as mastitis (1). Furthermore, MAP infection has been associated with a range of inflammatory and autoimmune conditions, including Crohn´s disease (4–6), type I diabetes (7), sarcoidosis (8), rheumatoid arthritis (9), Hashimoto’s thyroiditis (10), Blau syndrome (11) and multiple sclerosis (12). More recently, associations have also been suggested with other human disorders such as Alzheimer’s disease (13) and colorectal cancer (14).

Cattle are exposed to MAP via the fecal–oral route during the first months of life, through ingestion of contaminated feces, milk, water, or colostrum. Vertical transmission *in utero* has also been reported (15). Once ingested, MAP penetrates the intestinal mucosa through M cells located in Peyer’s patches, where it is phagocytosed by macrophages. Inside macrophages, MAP evades the host immune response through multiple mechanisms, including inhibition of macrophage apoptosis, prevention of phagosome acidification and subsequent fusion with lysosomes, suppression of antigen presentation via downregulation of the bovine *major histocompatibility complex II* (*BOLA*), and stimulation of the anti-inflammatory cytokine interleukin-10 (IL-10) (16). The mechanisms employed by MAP to evade immune clearance facilitate the establishment of chronic infection and significantly hinder the development of efficacious vaccines. PTB-associated lesions are classified as focal, multifocal, or diffuse, depending on the extent and cellular composition of the inflammatory infiltrate (17). Diffuse forms are further subdivided into three categories- lymphocytic/paucibacillary, intermediate, and multibacillary based on the nature of the infiltrate and the number of acid-fast bacilli present (17). In some infected animals, the granulomatous infiltrate becomes diffuse disrupting the intestinal mucosal structure. In advanced stages of the infection, MAP may also disseminate to the mammary gland, resulting in contamination of milk and colostrum (18, 19).

Granuloma formation is a key characteristic of MAP infection and is best described as an organized aggregate of various immune cells such as macrophages, multinucleated giant cells, epithelioid cells, lymphocytes, neutrophils, and fibroblasts. It allows these immune cells to enact a localized inflammatory response, encasing the bacilli within to prevent further dissemination, and inhibiting MAP replication. Focal and multifocal granulomas are typically associated with chronic and subclinical infection, whereas granulomas found in PTB-associated diffuse lesions are characteristics of disease progression and clinical disease. Recent studies suggest that host genetics, cellular composition, and the molecular mechanisms underlying PTB-associated multifocal and diffuse lesions may differ, and that progression from a multifocal to a diffuse lesion might not always occur (20–23). These previous studies have suggested that multifocal granulomas prevent lesions progression, but the molecular mechanisms involved in the establishment and maintenance of a chronic MAP infection are not fully understood.

**Figure 1:**
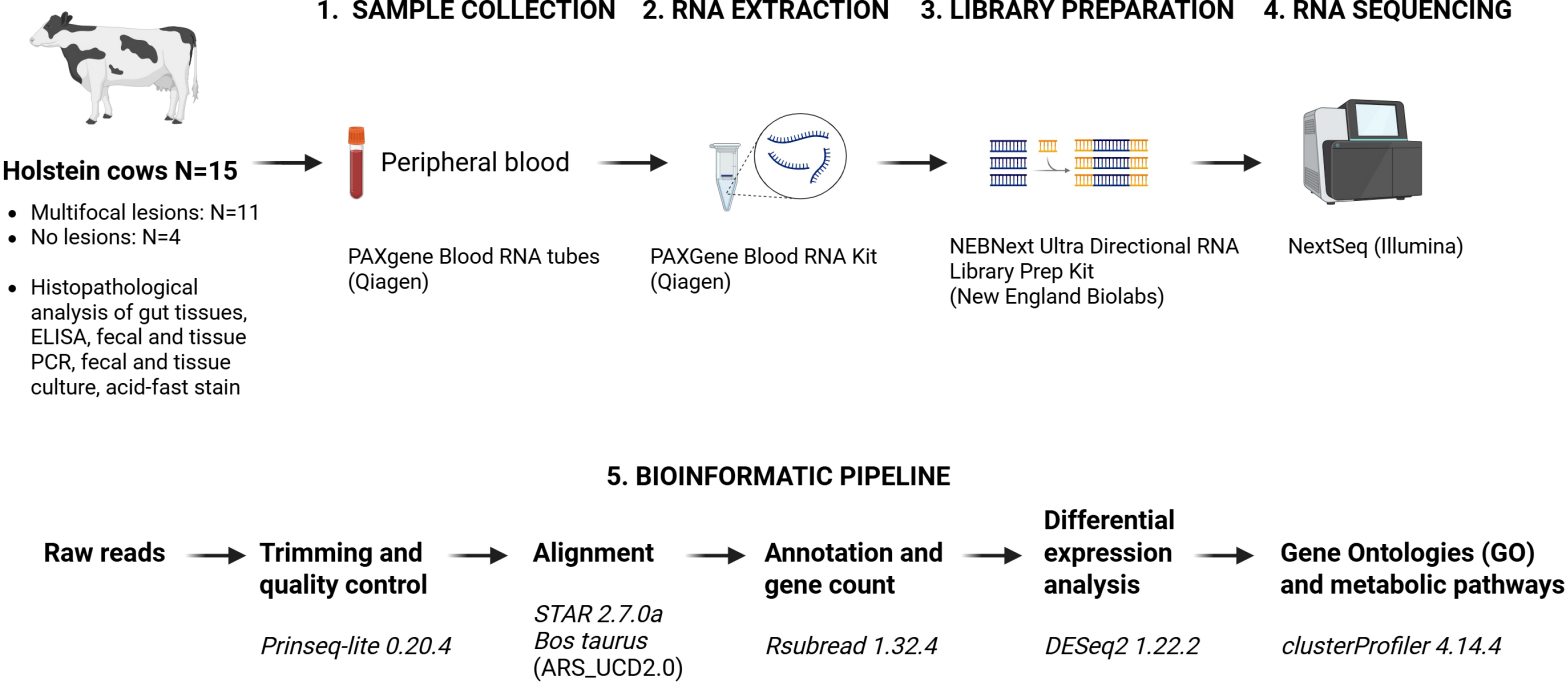
Study design. The infectious status of the animals included in this study was determined by histopathological analysis of gut tissues, acid-fast stain, ELISA for the detection of anti-MAP antibodies, and fecal and gut tissues PCR and bacteriological culture. Total RNA was extracted from peripheral blood samples from cows with multifocal lesions (N = 11) and control cows without lesions (N = 4). RNA libraries were prepared with the Illumina NEBNext Ultra Directional RNA library preparation kit and sequenced on an Illumina NextSeq sequencer. A bioinformatic pipeline was applied for differential expression analysis and the search for DEGs, GOs, and metabolic pathways. Created with BioRender.com.

Current diagnostic tests, MAP antibody ELISA and fecal PCR, do not allow the detection of all infected animals in a herd due to their low sensitivity for the animals in the subclinical stage of MAP infection. At this stage, MAP bacterial load is low and the host immune response is weak or delayed. These factors make conventional *ante-mortem* diagnostic methods insensitive, highlighting the need for novel biomarkers. Transcriptomic profiling is a source of novel biomarkers for the detection of animals in subclinical stages. RNA sequencing (RNA-Seq) is a third-generation sequencing tool that allows the complete transcriptomic analysis of a cell or tissue. RNA-Seq studies have used different samples from MAP-infected cattle including ileocecal valve (ICV) (22, 24–26), salivary glands (27), whole blood (22, 28), Peyer’s patches (29), jejunum (30, 31) and ileum (30). More recently, the ABCA13 transporter, a biomarker identified in peripheral blood (PB) from subclinically MAP-infected cattle, was successfully validated as a serum biomarker for detecting Holstein cows with focal lesions (22, 32). To date, no RNA-Seq studies have analyzed samples from MAP-infected animals exhibiting multifocal lesions in gut tissues. In this study, we performed RNA-Seq analyses of PB from cattle with PTB-associated multifocal lesions and from control animals without lesions. Gene expression data were analyzed using gene ontology and metabolic pathway enrichment to identify gene ontologies and metabolic pathways enriched for differentially expressed differentially expressed genes (DEGs) genes. Using the DEGs from the RNA-Seq data we performed protein-to-protein interaction analysis to identify functional networks and determine which proteins interact and act together. The workflow of the study is presented in **Figure 1**.

## 2 Materials and methods

### 2.1 Ethics statement

The study is reported in accordance with ARRIVE guidelines (https://arriveguidelines.org). The Animal Ethics Committee of the Servicio Regional de Investigación y Desarrollo Agroalimentario (SERIDA) approved the procedures on the animals included in this study. All procedures were authorized by the Regional Consejería de Agroganadería y Recursos Autóctonos of the Principality of Asturias (authorization codes PROAE 29/2015, PROAE 66/2019, and PROAE17/2022) and were carried out following the European Guidelines for the Care and Use of Animals for Research Purposes (2012/63/EU). PB, gut tissues, and fecal samples were collected by trained personnel and in accordance with good veterinary practice.

### 2.2 Animals and PTB diagnostic status

The animals included in this study came from a single commercial farm located in Asturias (Spain). The mean prevalence of PTB in the farm estimated by ELISA was 6,29% in the sampling period (2016–2018). The study population consisted of 15 Holstein cattle, 11 cows with PTB-associated multifocal lesions and 4 control cows without PTB-associated lesions in gut tissues. The infectious status of the animals included in this study was determined by histopathological analysis of gut tissues, acid-fast stain, ELISA for the detection of anti-MAP antibodies, and fecal and gut tissues PCR and bacteriological culture, as previously described (22). Only animals with well-characterized multifocal lesions and confirmed infection status (ZN, ELISA, fecal or tissue bacteriological culture and/or PCR) were included, while control animals (IDs 29–32) showed no PTB-associated lesions and were negative in all diagnostic tests. Without examining multiple tissue sites, infection could potentially be missed in the control cows. However, in our study, several tissue sites were analyzed histopathologically, including ileocecal lymph nodes, distal jejunal lymph nodes, ileocecal valve, and distal jejunum (22), to minimize this possibility. At the time of slaughter, none of the animals with multifocal lesions exhibited clinical signs of PTB, ensuring that this group represented a subclinical phenotype. The average age of animals without lesions and with multifocal lesions was 2.95 and 5.92 years old, respectively.

### 2.3 RNA extraction, library preparation and RNA-sequencing

At the time of slaughter, RNA was extracted from PB samples collected from the 15 cows included in the study. PB samples were collected from the coccygeal vein of cows using PAXgene Blood RNA tubes (2.5 ml) (Qiagen, Hilden, Germany). Total RNA was extracted with the PAXgene blood RNA kit according to manufacturer’s instructions (Qiagen, Hilden, Germany). Residual DNA was removed by digestion with RNase-free DNase I Amplification Grade following the recommended protocol (Invitrogen, Spain). The concentration and quality of the RNA were measured with the Agilent Bioanalizer 2100 (Agilent Technologies, Santa Clara, Ca, US). Only RNA samples with an RIN value ≥ 7 were selected for RNA-Seq (**Supplementary Table 1**). Each RNA library was prepared with approximately 250 ng of RNA using the Illumina NEBNext^®^ Ultra Directional RNA library preparation kit following the manufacturer`s instructions (Illumina, San Diego, CA, US). The RNA-Seq libraries’ quality was assessed on the Agilent Bioanalizer using a highly sensitive DNA chip to confirm that the insert size was between 188–274 bp for all individual libraries. All RNA-Seq libraries were quantified using a Qubit^®^ Fluorometer and doubled stranded DNA high Sensitivity Assay Kit (Invitrogen, Spain). Libraries were sequenced by single-end sequencing in 1x100 format on the Illumina NextSeq sequencer at the Genomic Unit of the Scientific Park of Madrid, Spain.

### 2.4 Bioinformatic analysis of RNA-Seq data

Raw reads were trimmed and filtered based on a Phred quality score >30, read length >100 bp, and a percentage of ambiguous bases <10% using Prinseq-lite 0.20.4 (33). Trimmed and filtered reads were subsequently aligned to the *Bos taurus* reference genome (ARS_UCD2.0) using the Spliced Transcripts Alignment to a Reference aligner (*STAR* 2.7.0a) (34). Reads were assigned to a gene if they were not multi-hit reads. The resulting aligned reads were used to generate a table of counts for each gene using the *FeatureCounts* function from the *R* library *Rsubread* 1.32.4 (35). Gene counts were then normalized with the mean-of-ratios method included in the *DESeq2* 1.22.2 software (36). Differential expression analysis of mRNAs for the comparison of cows with multifocal lesions *versus* (*vs*) controls was performed with *DESeq2*. An mRNA was considered differentially expressed (DE) if its false discovery rate (FDR)-adjusted P-value was ≤0.05 after correction for multiple testing using the Benjamini–Hochberg method (37).

### 2.5 Gene ontology and metabolic enrichment analysis

Genes differentially expressed between the animals with multifocal lesions and controls were analyzed for enrichment of gene ontologies (biological process, cellular components, molecular functions) and Kyoto Encyclopedia of Genes and Genomes (KEGG) pathways using the *R* library *ClusterProfiler* 4.14.4 (38). The Benjamini–Hochberg method was applied to adjust for multiple testing, considering a FDR-adjusted P-value ≤ 0.05 as significant. Only enriched metabolic pathways containing more than two DEGs were considered.

### 2.6 Protein to protein interaction networks

Using the DEGs with fold change (log_2_) ≥ or ≤ 2, PPI networks were analyzed using *String* 12.0 (39), setting the minimum required interaction score to 0.4. A STRING network is made up of nodes (proteins) and edges (interactions between two proteins). Proteins that participate in the same biological pathway typically form dense clusters, whereas proteins with few or no known interactions appear as isolated nodes. Proteins connecting different clusters are often key regulators or hub nodes. The networks were exported to *Cytoscape* 3.10 (40). for visualization of the level of expression of each gene in the network. The candidate proteins with no associations to other proteins were hidden.

### 2.7 Reverse transcription quantitative PCR for RNA-seq validation

Changes in Interferon stimulated gene 15 (ISG15) expression were validated by RT-qPCR using RNA isolated from the same animals analyzed by RNA-Seq. First-strand cDNA was synthesized from 250 ng of total RNA using the High-Capacity RNA to cDNA Kit (Applied Biosystems, Thermo Fischer) according to the manufacturer´s instructions. For the RT reactions, a total volume of 10 µl was incubated for 60 min at 42 °C to perform the RT and 5 min at 95 °C to inactivate the reverse transcriptase. RT control reactions without the enzyme were included. The resultant cDNAs were amplified using a ISG15 specific TaqMan Gene Expression Assay in a QuantStudio™ 12K Flex Real-Time PCR System (Applied Biosystems, Thermo Fisher). PCR conditions were: 50 °C for 2 min (UNG activation), 95 °C for 10 min (enzyme activation) followed by 40 cycles of 95 °C for 15 s (denaturation) and 60 °C for 60 s (annealing and extension). Appropriate controls (no template) were included. RT-qPCR experiments were performed by triplicate.

To obtain Ct values, the 2nd derivative max method of QuantStudio™ 12K Flex Software was used. Using the results of the samples from cows with multifocal lesions and without lesions, fold changes in expression were calculated using the 2^–ΔΔCt^ method. Normalization was performed using the *Glyceraldehyde 3-phosphate dehydrogenase (GAPDH)* reference gene. The results were expressed as fold change and were standardized by log_2_ transformation to be comparable to the RNA-Seq differential expression results. To test if the differences between the groups were statistically significant, an unpaired t-test was run using *R*. The Pearson correlation coefficient between the RNA-Seq and RT-qPCR quantitative results was calculated using *R*. Differences and correlations were considered statistically significant if the *P*-value was ≤ 0.05.

### 2.8 Bovine interferon stimulated gene 15 ELISA

The ISG15 expression was validated by quantitative sandwich ELISA in plasma samples (50 µl) from 18 animals without lesions and 25 with multifocal lesions according to the manufacturer’s instructions (MyBioSource, San Diego, US). The control cows tested negative by ELISA, PCR, and bacteriological culture of gut tissues. The sensitivity of the kit is 10 pg/ml and the detection range is 62.5 pg/ml-2000 pg/ml. Briefly, standards and samples (50 µl) were added in duplicate into an appropriate ELISA plate coated with the anti-ISG15 antibody. One hundred microliters of the horseradish peroxidase-conjugated antibody were added to each well. After incubation for 60 min at 37 °C, the plate was washed four times with 350 µl of wash solution and incubated with 50 µl of 3, 3′, 5, 5′-Tetramethylbenzidine for 15 min at 37 °C in the dark. After adding 50 µl of stop solution into each well, the OD values were measured in an ELISA reader at 450 nm (Thermo Scientific Multiskan, US). To obtain the final value for each standard and sample, the average OD of the blank wells was subtracted from their respective average OD values. A standard curve was generated by plotting the mean OD values of each standard on the vertical axis and the corresponding concentration on the horizontal axis. The concentration level of the ISG15 in each sample was interpolated from the standard curve. Statistical analysis was performed using an unpaired t-test for comparison between two groups (GraphPad Prism 8, San Diego, California, US). Differences were considered significant when P-value ≤ 0.05.

## 3 Results

### 3.1 Peripheral blood transcriptomic profiling of cattle with multifocal lesions

The infectious status of the animals included in this study (**Table 1**) was determined by histopathological analysis of gut tissues, acid-fast stain, ELISA for the detection of anti-MAP antibodies, and fecal and gut tissues PCR and bacteriological culture, as previously described (22). The cows with multifocal lesions were Ziehl–Neelsen (ZN) positive (10/11), gut tissue PCR positive (7/11), fecal PCR positive (4/11), tissue culture positive (4/11), fecal culture positive (2/11), and ELISA positive (1/11). Only one animal with multifocal lesions (ID 26) tested positive across all diagnostic methods, including ELISA, and showed a high bacterial load (>50 CFU/g) in gut tissues.

**Table 1:** Histopathological analysis, ZN stain, ELISA, PCR and bacteriological culture, from all the animals included in the study.

ID	Histological group	ZN	ELISA (OD)	Fecal PCR	Fecal culture (CFUs)	Tissue PCR	Tissue culture (CFUs)
18	Multifocal	Pos	Neg (3.46)	Nc	Neg	Pos	Neg
19	Multifocal	Neg	Neg (2.96)	Neg	Neg	Pos	Pos (10-50)
20	Multifocal	Pos	Neg (3.407)	Pos	Neg	Neg	Pos (10-50)
21	Multifocal	Pos	Neg (4.57)	Neg	Neg	Neg	Neg
22	Multifocal	Pos	Neg (4.58)	Neg	Neg	Pos	Neg
23	Multifocal	Pos	Neg (5.06)	Neg	Neg	Pos	Neg
24	Multifocal	Pos	Neg (12.56)	Pos	Pos (>50)	Pos	Neg
25	Multifocal	Pos	Neg (1.65)	Neg	Neg	Neg	Neg
26	Multifocal	Pos	Pos (67.58)	Pos	Pos (<10)	Pos	Pos (>50)
27	Multifocal	Pos	Neg (13.72)	Pos	Neg	Pos	Neg
28	Multifocal	Pos	Neg (1.50)	Neg	Neg	Neg	Pos (<10)
29	Without lesions	Neg	Neg (2.91)	Neg	Neg	Neg	Neg
30	Without lesions	Neg	Neg (2.95)	Neg	Neg	Neg	Neg
31	Without lesions	Neg	Neg (1.26)	Neg	Neg	Neg	Neg
32	Without lesions	Neg	Neg (2.45)	Neg	Neg	Nc	Neg

Neg, Negative; Pos, Positive, Nc; non-conclusive; ZN, Ziehl-Neelsen; OD, Optical density; CFU, colony forming units.

RNA-Seq libraries were prepared from PB samples of 11 cows with PTB-associated multifocal histopathological lesions and 4 control cows without lesions in gut tissues. The RNA-Seq data summary for each biological sample including raw reads and uniquely mapped reads is provided in **Supplementary Table 1**. After quality control, an average of 34.08 million filtered reads were retained. Alignment of these reads to the *Bos taurus* reference genome yielded an average of 31.22 (91%) million mapped reads per library. Of the mapped reads, an average of 2.57 million reads (8%) aligned to multiple genomic locations and were excluded from gene expression analysis. Reads mapped to unique genomic locations were analyzed in detail using *FeatureCounts*, a software package designed to quantify reads in 3’ UTR and 5’ UTR, exons, introns and intergenic regions. Analysis of PB reads mapped to unique genomic locations revealed that 24.34% aligned to exons, 66.48% to introns, 9.18% to intergenic regions, 13.96% to 3’ UTRs and 1.22% to 5’ UTRs. The resulting aligned reads were used to generate a table of counts for each gene. Gene counts normalized with the mean-of-ratios method included in the *DESeq2* 1.22.2 software are presented in **Supplementary Table 2**.

### 3.2 Identification of differentially expressed genes in PB from cows multifocal lesions *vs* control cows

**Figure 2:**
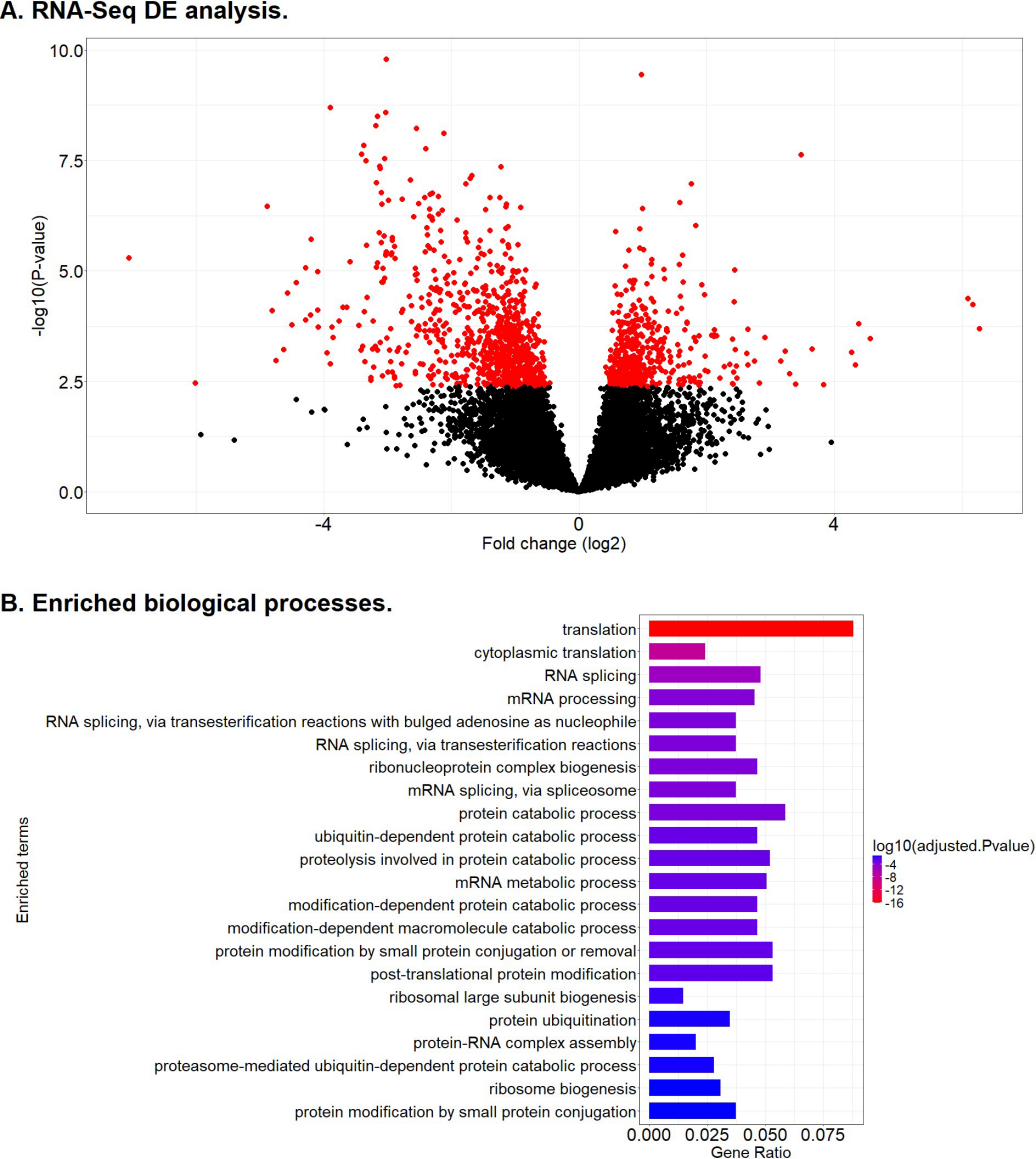
RNA-Seq DE analysis. **(A)** Volcano plots showing the DE fold change (log_2_) *vs* -log (P-value). The red spots represent the DEGs (N = 1272) between cows with multifocal lesions *vs* controls. **(B)** Enriched biological processes based on DEGs in animals with multifocal lesions compared to controls.

Differential expression analysis of mRNAs comparing cows with multifocal lesions *vs* controls was performed with *DESeq2*. Genes differentially expressed in PB samples (FDR-adjusted P-value ≤ 0.05) are highlighted as red dots in **Figure 2A**. Among the 1,272 genes differentially expressed in PB samples from cows with multifocal lesions compared to controls, 442 were upregulated and 830 downregulated. **Table 2** shows the top upregulated genes (fold change (log_2_) > 2) in PB from cows with multifocal lesions *vs c*ontrols. Among the top 15 genes in the PB samples were *the Cytochrome P450 Family 4 Subfamily F Member 22* (*CYP4F22*; FDR-adjusted P-value = 6.98E-03), *Adenylate Cyclase 8* (*ADCY8*; FDR-adjusted P-value = 1.66E-02), *Latent Transforming Growth Factor Beta Binding Protein 4 (LTBP4*; FDR-adjusted P-value = 4.69E-02), *T-Box Transcription Factor 3 (TBX3;* FDR-adjusted P-value = 1.49E-02), and *2’-5’-Oligoadenylate Synthetase 1 (OAS1Y;* FDR-adjusted P-value = 1.05E-02). All these genes were upregulated, with fold changes ranging from 2.9-6.3).

**Table 2:** DEGs (fold change (log_2_) > 2) in PB samples from cows with multifocal lesions *vs* controls.

Gene ID	Description	Fold change (Log_2_)	FDR-adjusted p-value
ENSBTAG00000055484	Uncharacterized protein	6.28	8.12E-03
ENSBTAG00000073693	Uncharacterized protein	6.17	3.88E-03
ENSBTAG00000059510	Uncharacterized protein	6.10	3.12E-03
ENSBTAG00000077237	Uncharacterized protein	4.57	1.08E-02
ENSBTAG00000059781	Cytochrome P450 Family 4 Subfamily F Member 22 (*CYP4F22*)	4.39	6.98E-03
ENSBTAG00000078470	Uncharacterized protein	4.33	2.50E-02
ENSBTAG00000014600	Adenylate Cyclase 8 (*ADCY8*)	4.27	1.66E-02
ENSBTAG00000075045	Uncharacterized protein	3.84	4.74E-02
ENSBTAG00000021706	T-Box Transcription Factor 3 (*TBX3*)	3.65	1.49E-02
ENSBTAG00000056982	Uncharacterized protein	3.48	3.03E-05
ENSBTAG00000004757	Latent Transforming Growth Factor Beta Binding Protein 4 (*LTBP4*)	3.40	4.69E-02
ENSBTAG00000075138	Uncharacterized protein	3.31	3.32E-02
ENSBTAG00000078609	Uncharacterized protein	3.23	1.58E-02
ENSBTAG00000050199	Uncharacterized protein	3.17	2.20E-02
ENSBTAG00000039861	2’-5’-Oligoadenylate Synthetase 1 (*OAS1Y*)	2.92	1.05E-02
ENSBTAG00000011131	Neuromedin U Receptor 2 (*NMUR2*)	2.83	4.57E-02
ENSBTAG00000006037	Cellular Communication Network Factor 5 (*CCN5*)	2.75	2.19E-02
ENSBTAG00000003776	Myb/SANT DNA Binding Domain Containing 1 (MSANTD1)	2.65	2.49E-02
ENSBTAG00000013798	Protein Tyrosine Phosphatase Receptor Type N (*PTPRN*)	2.65	8.18E-03
ENSBTAG00000050515	Uncharacterized protein	2.63	1.69E-02
ENSBTAG00000073538	Uncharacterized protein	2.48	3.83E-02
ENSBTAG00000013867	DLG Associated Protein 3 (*DLGAP3*)	2.47	2.59E-02
ENSBTAG00000076331	Uncharacterized protein	2.47	2.61E-02
ENSBTAG00000078567	Uncharacterized protein	2.45	1.51E-02
ENSBTAG00000037404	Leucine Rich Repeat And Fibronectin Type III Domain Containing 4 (*LRFN4*)	2.45	1.23E-03
ENSBTAG00000022927	Rac Family Small GTPase 3 (*RAC3*)	2.43	3.47E-03
ENSBTAG00000014707	Interferon-Induced 15 KDa Protein (*ISG15*)	2.42	3.19E-02
ENSBTAG00000005734	GATA Binding Protein 6 (*GATA6*)	2.42	4.69E-02
ENSBTAG00000076291	Uncharacterized protein	2.41	4.57E-02
ENSBTAG00000015557	Uncharacterized protein	2.40	1.09E-02
ENSBTAG00000055358	Uncharacterized protein	2.37	2.44E-02
ENSBTAG00000000936	Solute Carrier Family 39 Member 2 (*SLC39A2*)	2.28	2.65E-02
ENSBTAG00000015350	Perilipin 1 (*PLIN1*)	2.21	3.82E-02
ENSBTAG00000012406	Z-DNA Binding Protein 1 (*ZBP1*)	2.16	1.00E-02
ENSBTAG00000046580	DExH-Box Helicase 58 (*DHX58*)	2.13	9.68E-03
ENSBTAG00000014693	Transmembrane Protein 88 (*TMEM88*)	2.12	8.32E-03
ENSBTAG00000051030	Cytochrome P450 family 2 subfamily J member 30 (*CYP2J30*)	2.11	1.02E-02
ENSBTAG00000035998	Creatine Kinase B (*CKB*)	2.08	9.91E-03
ENSBTAG00000003079	Tigger Transposable Element Derived 3 (*TIGD3*)	2.02	3.13E-02

### 3.3 Functional enrichment analysis of differentially expressed genes in PB from cows with multifocal lesions *vs* control cows

Genes with differential expression between the animals with multifocal lesions *vs* controls were investigated for the enrichment of gene ontologies (biological process, cellular components, molecular functions) using *clusterProfiler*. All the molecular functions, biological processes, and cellular components that were enriched in the PB samples of the cows with multifocal lesions *vs* controls are summarized in **Supplementary Table 3**. A total of 22 biological processes were enriched in the PB samples from animals with multifocal lesions *vs* controls, respectively (**Figure 2B**). The top-10 enriched GOs were associated with translation (GO:0006412 and GO:0002181), RNA splicing (GO:0008380, GO:0000375, GO:0000377, GO:0000398), mRNA processing (GO:0006397), ribonucleoprotein complex biogenesis (GO:0022613), and protein catabolic process (GO:0030163, GO:0006511), among others. In agreement with these data, some of the enriched cellular components were related to the ribosome (GO:0022626, GO:0005840), ribonucleoprotein (GO:1990904), and spliceosomal complex (GO:0005681).

### 3.4 Pathway enrichment analysis of DEGs in peripheral blood from cows with multifocal lesions *vs* control cows

Using the DEGs identified in PB, enrichment pathway analysis was performed with *Cluster Profiler*. As shown in **Table 3**, four metabolic pathways referenced in the KEGG database were significantly enriched in the PB samples from cows with multifocal lesions *vs* controls, including the ribosome (bta03010, FDR-adjusted P-value = 1.85E-22), COVID-19 (bta05171, FDR-adjusted P-value = 1.85E-19), thermogenesis (bta04714, FDR-adjusted P-value = 2.18E-02) and Parkinson disease (bta05012, FDR-adjusted P-value = 3.51E-02). The ribosome pathway was enriched for 35 differentially expressed ribosomal protein (RP) genes, which were consistently downregulated, suggesting a potential global reduction in translational activity in cows with multifocal lesions relative to controls. Notably, these same RP genes were also included in the COVID-19 pathway.

**Table 3:** Enriched metabolic pathways in PB samples from cows with multifocal lesions *vs* control cows.

Description	ID	FDR-adjusted p-value	Gene ID
Ribosome	bta03010	1.85E-22	*MRPS2/RPS11/RPL12/RPL4/RPL6/RPL32/RPL14/RPS2/RPL13A/RPL35/RPL21/RPLP0/RPL27/RPL5/FAU/MRPL1/RPL22L1/RPS8/RPS7/LOC101907658/RPS20/LOC101902907/RPL30/LOC101906221/RPL34/RPL11/RPL9/LOC101905894/LOC107132967/RPL17/LOC101906914/RPS15A/LOC619131/RPL37A/LOC132344361/RPS3A/RPL26/RPS25/RPS27/RPL23/RPL39/LOC101902490/LOC101907518/LOC112441655/LOC132346402/RPL36A/RPL37/RPL35A/RPS24/LOC781576/RPL36AL/RPL22*
COVID-19	bta05171	5.18E-19	*OAS1Y/ISG15/MX1/OAS2/C2/C3/CFD/IRF9/NFKBIB/RPS11/RPL12/RPL4/RPL6/RPL32/RPL14/RPS2/RPL13A/MAP3K7/RPL35/RPL21/RPLP0/RPL27/RPL5/FAU/RPL22L1/RPS8/RPS7/LOC101907658/RPS20/LOC101902907/RPL30/LOC101906221/RPL34/RPL11/RPL9/LOC101905894/LOC107132967/RPL17/LOC101906914/RPS15A/LOC619131/RPL37A/LOC132344361/RPS3A/RPL26/RPS25/RPS27/RPL23/RPL39/LOC101902490/LOC101907518/LOC112441655/LOC132346402/RPL36A/RPL37/RPL35A/RPS24/LOC781576/RPL36AL/RPL22*
Thermogenesis	bta04714	2.18E-02	*ADCY8/PLIN1/SMARCB1/RPS6KA1/ATP5F1B/NDUFA9/NDUFA10/KDM3A/ATF2/KRAS/PRKAA1/NDUFB4/FRS2/KLB/NDUFS5/COX7A2/NDUFA5/NDUFB3/UQCRH/COX7B/ATP5F1E/LOC132343467/COX7C/LOC101902937/COX6C/LOC132344161*
Parkinson disease	bta05012	3.51E-02	*SLC39A2/SLC39A5/ATP5F1B/NDUFA9/PSMC5/ADRM1/NDUFA10/KEAP1/UBE2J1/ITPR2/PSMA2/PSMC6/NDUFB4/SLC39A10/NDUFS5/PSMA3/COX7A2/NDUFA5/NDUFB3/UQCRH/COX7B/TUBA3E/ATP5F1E/LOC132343467/COX7C/LOC101902937/COX6C/LOC132344161*

### 3.5 Protein–to-protein interaction networks revealed coordinated translation inhibition and innate immune activation

**Figure 3:**
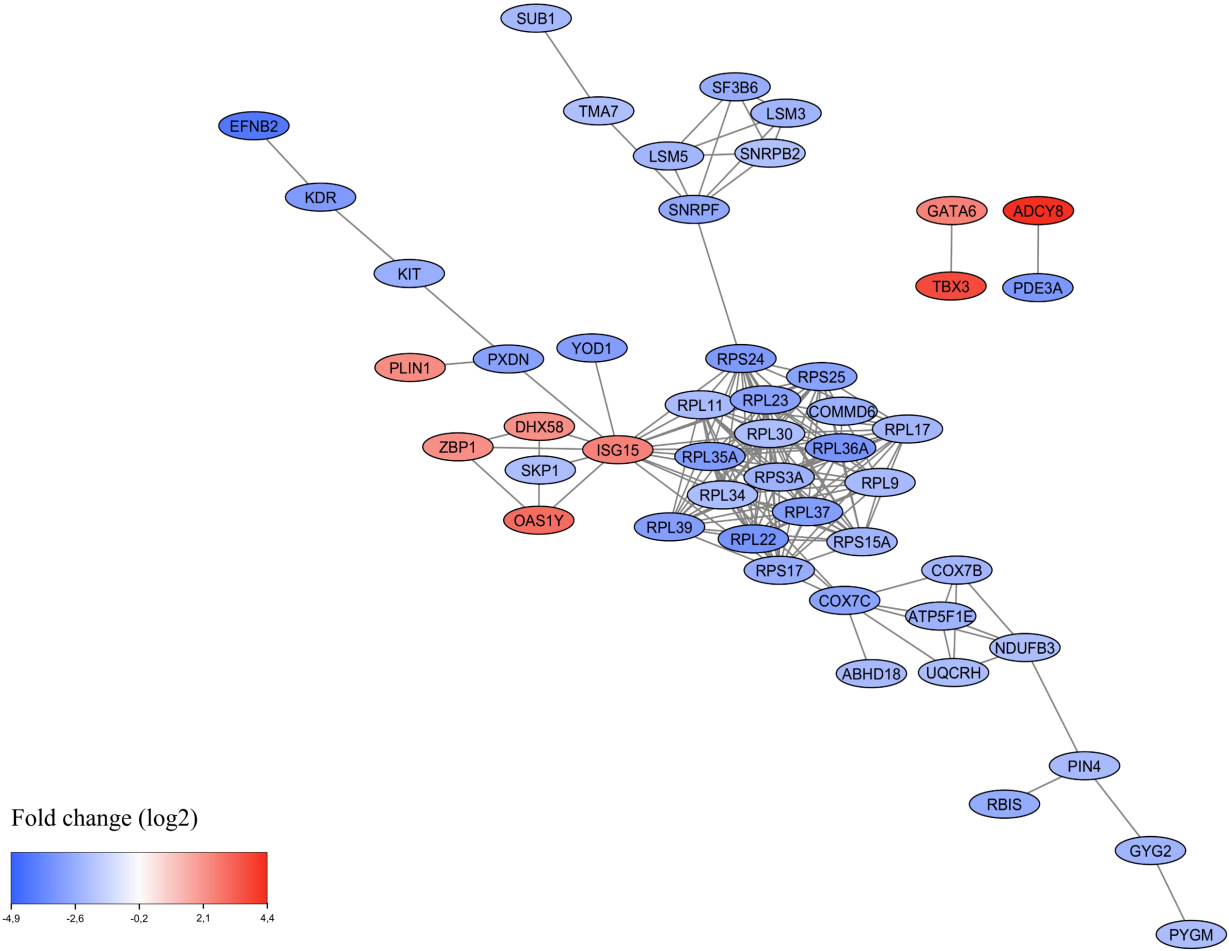
Protein-protein network analysis using the DEGs [fold change (log_2_ ≥ 2 and ≤ 2)] in blood samples from cows with multifocal lesions *vs* the control group. Individual nodes represent proteins with relationships represented by edges. The proteins with no associations to other proteins in the networks were hidden.

PPI networks analysis of DEGs (fold change (log_2_) ≥ 2 or ≤ 2) from cows with multifocal lesions *vs* controls was performed using *STRING.* The analysis identified atotal of 131 nodes, 861 edges, 2 functional associations and 1 network with significant PPI enrichment (P-value:< 1.0E-16). Using *Cytoscape*, upregulated and downregulated genes in the PPI network were represented in red and blue colors, respectively. As shown in **Figure 3**, the network contains a central hub composed of 17 downregulated RPs involved in translation. A functional interaction between the *RPS24* and the *Small Nuclear Ribonucleoprotein Polypeptide F* (*SNRPF*; fold change = –2.76; FDR-adjusted P-value = 1.19E-04), involved in spliceosomal snRNP assembly, suggests inhibition not only of translation but splicing as well in the animals with multifocal lesions. Interestingly, functional associations of four upregulated genes that trigger activation of the innate immune response including *ISG15* (fold change = 2.42; FDR-adjusted P-value = 3.19E-02), a DNA-Dependent activator of IFN-Regulatory Factors (*ZBP1;* fold change = 2.16; FDR-adjusted P-value = 1.00E-02), *DExH-Box Helicase 58* (*DHX58;* fold change = 2.13; FDR-adjusted P-value = 9.68E-03), and *OAS1Y* (fold change = 2.91; FDR-adjusted P-value = 1.05E-02) was observed. *ISG15*, through ISGylation, can modify ribosomal proteins and translation factors, and drives a controlled shutdown of global translation (41).

### 3.6 Validation of the *ISG15* mRNA differential expression in PB samples by RT-qPCR

**Figure 4:**
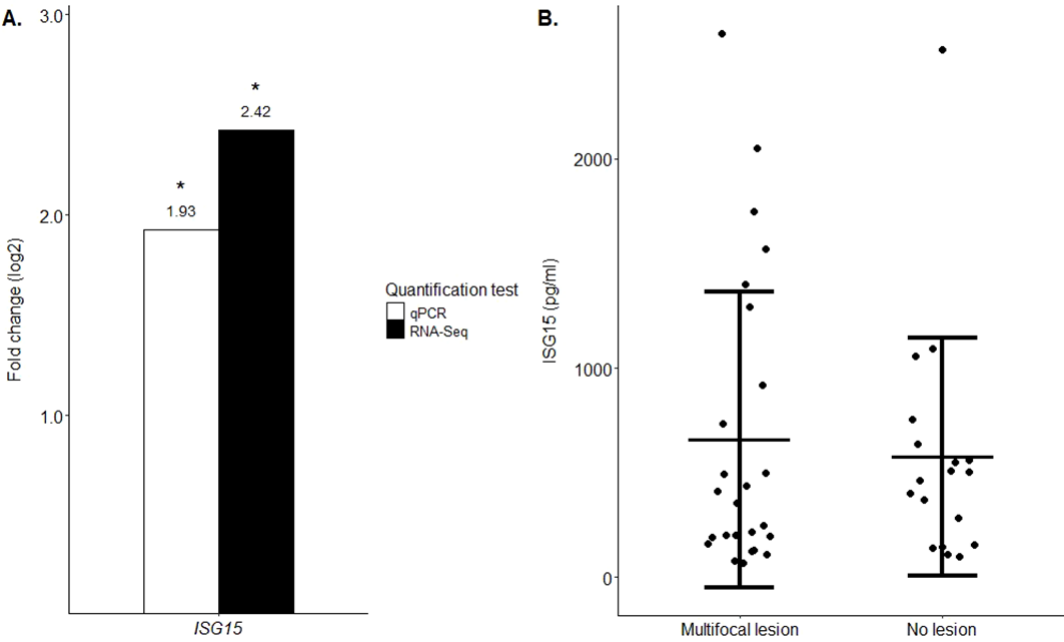
Differential expression of ISG15 in PB samples analyzed by RT-qPCR and ELISA. **(A)** Validation of the *ISG15* mRNA differences in expression performed by RT-qPCR. Changes in the expression levels, expressed as the fold change (log_2_), were calculated using RNA-Seq (in black) and RT-qPCR (in white). Significant changes in mRNA expression (P ≤ 0.05) are indicated with an asterisk. **(B)** Quantitative sandwich ELISA was used to measure ISG15 protein in plasma samples from cows without lesions and PCR, ELISA and bacteriorological culture negative (control, N = 18) and cows with multifocal lesions (N = 25). Cows with multifocal lesions exhibited elevated mean plasma ISG15 levels (656.85 pmol/μl) compared with controls (575.29 pmol/μl). However, the difference (~81.5 pmol/μl) did not reach statistical significance (P-value = 0.6881). Data are presented as mean ± standard error of the mean (SEM). Each sample is represented by a circle on the dot plot.

Since *ISG15* mRNA was upregulated (fold change = 2.42) in cattle with multifocal lesions *vs* controls, appeared to connect different clusters within the PPI network, and may act as a key regulator of the observed transcriptional changes, we evaluated its potential as a biomarker. *ISG15* mRNA differential expression was validated by RT-qPCR using total RNA isolated from PB samples of the animals included in the study. The results of the qRT-PCR are presented in **Figure 4A** and were expressed as fold changes and standardized by log_2_ transformation to be comparable to the RNA-Seq differential expression results. The normalization of the RT-qPCR results was performed using the expression of the *GAPDH* as the endogenous reference gene. The RT-qPCR results showed a statistically significant upregulation of the *ISG15* mRNA in the comparison of cows with multifocal lesions *vs* controls (fold change = 1.93; P-value = 0.03). A similar trend (upregulation) was observed in the results obtained with both RNA-Seq and RT-qPCR methods. Indeed, the Pearson correlation coefficient calculated for the RNA-Seq and RT-qPCR fold change data was ρ= 0.98 (P-value = 1.667E-11).

### 3.7 Plasma ISG15 protein levels in animals with and without multifocal lesions

The ISG15 was tested by quantitative sandwich ELISA in plasma samples from 18 animals without lesions and with negative diagnostic test results and 25 animals with multifocal lesions. **Figure 4B** shows that cows with multifocal lesions had higher mean plasma ISG15 protein levels (656.85 pmol/μl) than control cows (575.29 pmol/μl). However, the difference (~81.5 pmol/μl) did not reach statistical significance (P-value = 0.6881).

## 4 Discussion

Transcriptomic profiling of the host responses to MAP infection has the potential to enhance our understanding of MAP–host interactions, elucidate mechanisms underlying the control or progression of MAP infection, and provide a valuable source of biomarkers for the development of novel diagnostic tools. Transcriptional responses in blood and tissue in response to MAP infection are known to differ due to specific cellular composition and immune dynamics (22). Intestinal tissues and draining lymph nodes reflect localized and often heterogeneous responses, which can limit the detection of consistent DEGs, particularly in early and subclinical stages of MAP infection. In contrast, PB captures systemic immune responses and circulating signals released from infected tissues, making blood transcriptomics more sensitive for detecting coordinated host responses. Moreover, focusing on blood transcriptomics allows the identification of systemic biomarkers in cattle during the subclinical stage of MAP infection. To our knowledge, this study is the first to compare PB gene expression profiles of naturally MAP infected cattle with multifocal lesions (N = 11) to uninfected control cows (N = 4) using RNA-Seq. In a herd where MAP is present, most animals have been exposed to the pathogen, making it challenging to identify uninfected controls. As a result, control cows in such herds are often limited in number and may not be perfectly age-matched with infected cows. In this study, controls were fewer in number compared with the multifocal lesion group. However, all animals came from the same herd, ensuring comparable environmental exposure. For genes with a large effect size (e.g., 2-fold change), a group of 4 controls versus 11 lesion-positive cows provides high statistical power. However, power may decrease for moderate or small fold changes (42). The unbalanced study design may also affect variance estimates, increasing the likelihood of false negatives, particularly for genes with moderate expression changes; therefore, non-significant trends should be interpreted cautiously. However, *DESeq2*, used in this study for differential expression analysis, mitigates some of these limitations by stabilizing dispersion and fold-change estimates, even when group sizes are small or unbalanced. Unlike previous studies, which primarily selected RNA-Seq samples from animals (uninfected, clinical and subclinical) based on ELISA and/or fecal PCR results, our RNA-Seq study specifically selected naturally MAP-infected animals with multifocal lesions and without clinical signs, allowing identification of early host responses that were often missed in earlier studies. Comparing RNA-Seq studies is challenging due to differences in experimental design, tissue type, infection stage, and sample preparation, as well as variability in sequencing platforms, library preparation, read depth, and bioinformatics pipelines. Differences in statistical thresholds, genome annotations, and normalization methods can further complicate comparisons.

In this study, PB samples were selected for RNA-Seq analysis to capture systemic host responses to MAP infection. Comparison of cows with multifocal lesions to controls, revealed 1,272 DEGs in PB, including both 442 upregulated and 830 downregulated genes. These findings are consistent with previous transcriptomic studies in subclinical PTB, which also reported significant systemic transcriptional changes detectable in blood (28). Analysis of the top 15 upregulated genes in PB samples indicates that cattle with multifocal lesions exhibit upregulation of anti-inflammatory molecules (*LTBP4*, *TBX3*, *ADCY8*), suggesting a regulatory response aimed at limiting tissue damage. At the same time, immune surveillance remains active but tightly regulated (*OAS1Y*), while the observed upregulation of *CYP4F22* may reflect a balance between host defense and pathogen persistence, possibly by modulating lipid-mediated immune responses. The *LTBP4* (fold change = 3.4; FDR-adjusted P-value = 4.69E-02), is a key regulator of *transforming growth factor beta* (*TGF-ß*) that controls TGF-ß activation by maintaining it in a latent state during storage in extracellular space (43). The *OAS1Y* (fold change = 2.9; FDR-adjusted P-value = 1.05E-02) was also highly upregulated in PB samples from cows with multifocal lesions *vs* controls. OAS1Y, belonging to 20-50-oligoadenylate synthetases (OAS), is a dsRNA-activated antiviral enzyme which plays a critical role in cellular innate immune response and cellular processes like cell growth, apoptosis, positive regulation of Interferon-ß (IFN-ß) production, and tumor necrosis factor (TNF) production (44). Polymorphisms in the *OAS1Y* gene have been associated with increased OAS1 levels and reduced susceptibility to viral infections, including SARS-CoV-2 and Type-1 diabetes mellitus (45–47). Among its related pathways are antiviral mechanism by IFN-stimulated genes (GO:0032728) and host-pathogen interaction of human coronaviruses-IFN induction (GO:0032760). In addition, it has been previously reported that the expression of *OAS1*, *OAS2* and *OAS3* in human macrophages infected with *Mycobacterium tuberculosis* restricts mycobacterial intracellular replication and enhance cytokine secretion (48). In our DE analysis, we found that *OAS2* (fold change = 1.89; FDR-adjusted P-value = 3.55E-02) was also upregulated in PB samples from animals with multifocal lesions *vs* controls.

The PPI analysis revealed functional associations among genes involved in the innate immune response that were upregulated in PB samples from cattle with multifocal lesions compared with controls, including *OASIY*, *ISG15* (fold change = 2.42; FDR-adjusted P-value = 3.19E-02), *DHX58* (fold change = 2.12; FDR-adjusted P-value = 9.68E-03), and *ZBP1* (fold change = 2.16; FDR-adjusted P-value = 1.00E-02). The upregulation of *ISG15, ZBP1, DHX58*, and *OAS1Y* aligns with prior evidence that MAP infection triggers innate immune activation in subclinical or early-stage PTB (49, 50). ISG15 is a ubiquitin-like protein that plays a key role in the activation of the innate immune response either via its conjugation to a target protein (ISGylation) or via its action as a free or unconjugated protein (51). Through ISGylation, ISG15 triggers activation of innate immunity against a range of viruses, including coronaviruses, flaviviruses and picornaviruses, blocking the entry, replication, trafficking or release of intracellular pathogens. Apart from the conjugated form, the secreted form of ISG15 can induce natural killer cell proliferation, function as a chemotactic factor for neutrophils and act as an IFNγ-inducing cytokine playing an essential role in antimycobacterial immunity (52). The tumor stroma and activated macrophage protein (*ZBP1*) is an innate immune sensor that regulates cell death and inflammation. It contributes to the innate immune response by binding to foreign DNA and inducing Type-I IFN production. ZBP1 acts as an essential mediator of Pyroptosis, Necroptosis and Apoptosis (PANoptosis), an integral part of host defense against pathogens, by activating RIPK3, caspase-8 (CASP8), and the NLRP3 inflammasome. Finally, *DHX58* is involved in negative regulation of type I IFN production and regulation of innate immune response to various RNA viruses and some DNA viruses such as poxviruses and coronavirus SARS-CoV-2, and also to the bacterial pathogen *Listeria monocytogenes* (53, 54). Collectively, our results suggest that OAS1Y, ISG15, ZBP1, and DHX58 form a defense network that detects intracellular bacteria, activates and regulates innate immune responses, and restricts bacterial replication in animals with PTB-associated multifocal lesions. In the PPI analysis, we also observed that in PB samples from cattle with multifocal lesions, ISG15 interacts with a central hub composed of 17 downregulated RPs involved in translation. Due to the central position of ISG15 in the network, we evaluated its potential as a biomarker. The *ISG15* mRNA expression changes observed in PB samples by RNA-Seq were validated using RT-qPCR. Although *ISG15* was upregulated at the mRNA level in PB from cows with multifocal lesions, this increase was not statistically significative in plasma protein levels measured by ELISA. This discrepancy may be due to post-transcriptional regulation of *ISG15*, protein stability, secretion rates or extracellular degradation. RNA-Seq can detect even modest transcriptional changes because mRNA is amplified, while ELISA relies on absolute protein concentration.

**Figure 5:**
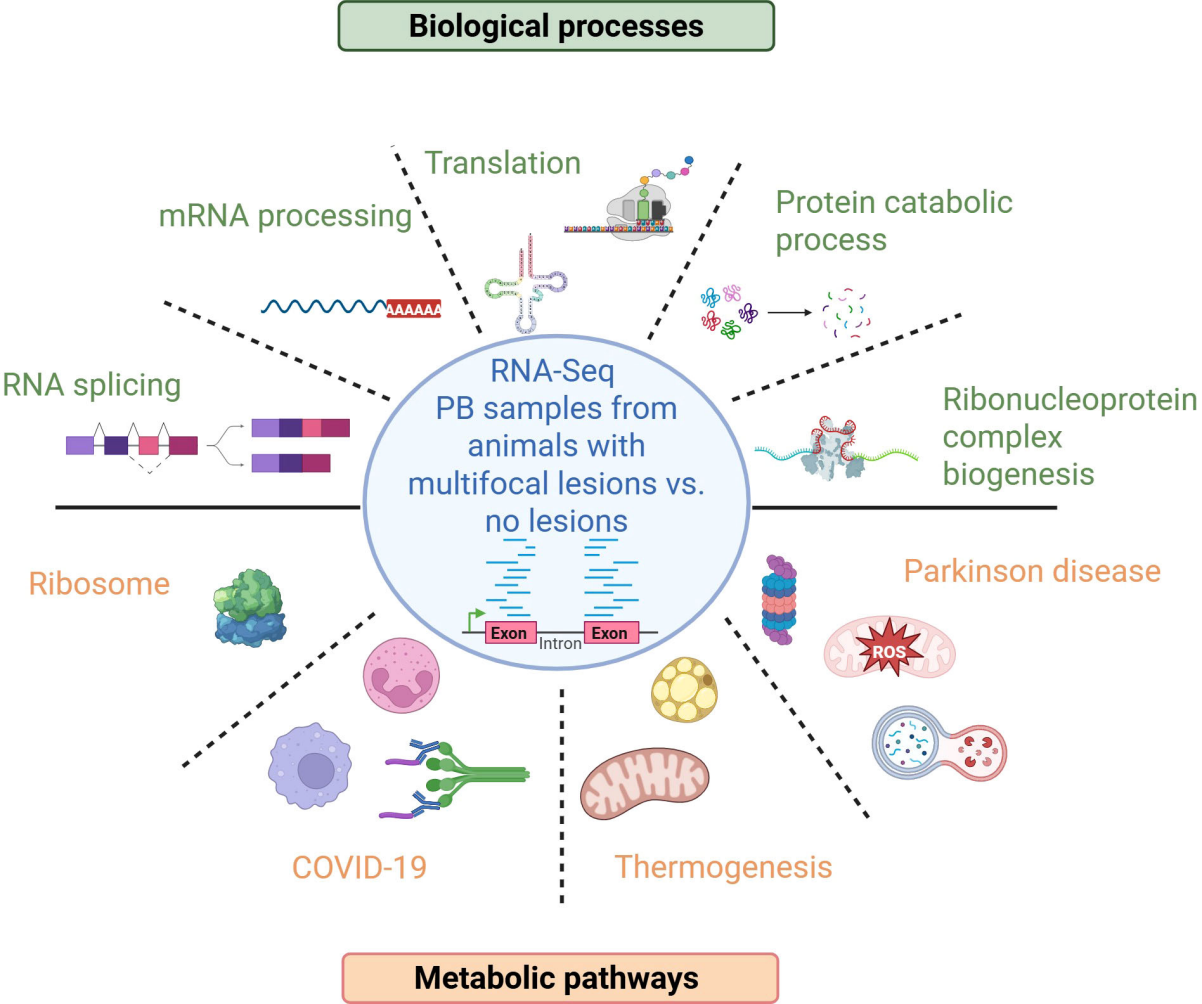
Enriched biological processes and metabolic pathways in PB of animals with multifocal lesions compared to control cows without gut lesions. The gene ontology terms shown in the figure were predominantly enriched among downregulated genes, suggesting that these biological processes are likely suppressed. Created with BioRender.com.

Our findings align with previous studies showing that ISG15, through the process of ISGylation, can modify ribosomal proteins and translation factors, resulting in a controlled reduction of global protein synthesis (55). Such translational regulation enables cells to prioritize the production of immune and stress-response proteins over general housekeeping proteins and serves as a valuable indicator of latent or stress-adapted cellular states. The observation of downregulated ribosomal protein genes and enrichment of translation-related GO terms is a novel finding in PTB research. While other chronic infections (e.g., tuberculosis) have shown translational inhibition in host cells (56), ours is one of the first studies to suggest systemic translational downregulation in subclinical MAP infection in cattle, potentially linked to controlled immune responses or pathogen-mediated modulation. Translational regulation plays a role in adaptation to stress and pathogen persistence, supporting the idea that modulation of translation is a conserved response in chronic infection contexts. In the PPI analysis, we also observed a functional interaction between the RPS24 (fold change = -3.15; FDR-adjusted P-value = 9.86E-06) and the Small Nuclear Ribonucleoprotein Polypeptide F (SNRPF) (fold change = -2.76; FDR-adjusted P-value = 1.19E-04), involved in spliceosomal snRNP assembly, which suggests inhibition not only of translation but splicing as well in the animals with multifocal lesions. In the PPI analysis, SNRPF was found to interact with other splicing-related molecules, including Small Nuclear Ribonucleoprotein Polypeptide B2 (SNRPB2; fold change = -2.02; FDR-adjusted P-value = 8.28E-03), U6 Small Nuclear RNA and mRNA Degradation Associated 3 (LSM3; fold change = -2.37; FDR-adjusted P-value = 5.69E-04), and LSM5 (fold change = -2.31; FDR-adjusted P-value = 2.21E-03), all of which were downregulated. Global disruption of mRNA splicing may decrease host protein and mRNA levels by triggering non-sense mediated decay of improperly spliced mRNAs (57). The results of the PPI analysis correlated with the enrichment of GO analysis since translation (GO:0006412), RNA splicing (GO:0008380), and mRNA processing (GO:0006397) were enriched in the PB samples of cow with multifocal lesions *vs* controls. The main GOs and pathways identified in PB of cattle with multifocal lesions *vs* controls are summarized in **Figure 5**. In line with these findings, the cellular components and pathways enriched in the analysis were primarily associated with the ribosome (GO:0022626, GO:0005840, bta03010), ribonucleoprotein complexes (GO:1990904), and the spliceosomal complex (GO:0005681). In a previous RNA-Seq study, no significant overexpressed GO terms were identified in PB of cattle with subclinical PTB and focal lesions (22). That study also revealed strong downregulation of *CXCL8/IL8* in PB and ICV of cattle with both focal and diffuse lesions. In the current study, *CXCL8/IL8* downregulation was again observed in PB (fold change = -2.10) suggesting a consistent suppression of neutrophil chemotaxis during MAP infection.

Enriched metabolic pathways in the comparison of cows with multifocal lesions *vs* controls included those related to COVID-19 (bta05171), thermogenesis (bta04714), and Parkinson’s disease (bta05012). The genes set shared between cows with multifocal lesions and COVID-19 reflects a coordinated response to both MAP and COVID-19 infection, capturing three major biological processes: IFN and complement mediated antiviral defense, and broad remodeling of host translation machinery. Several innate immune related genes, many of which are also associated with COVID-19, such as *OAS1Y, OAS2, ISG15*, *Interferon-Induced GTP-Binding Protein MX1*, *Complement C2, Complement C3, Complement factor D (CFD), Interferon Regulatory Factor 9 (IRF9)* appeared enriched in the transcriptome of cows with multifocal lesions. Regulators of inflammatory signaling, such as *NFKB Inhibitor Beta (NFKBIB;* fold change = 0.76; FDR-adjusted P-value = 3.73E-02) and *Mitogen-Activated Protein Kinase 7* (*MAP3K7;* fold change = -1.13; FDR-adjusted P-value = 1.89E-03), were also enriched and might restrain excessive inflammatory signaling. Many of the genes in the list are downregulated ribosomal protein genes (*RPL* and *RPS* family members) reflecting the extensive translational reprogramming that occurs in infected cattle with multifocal lesions. As observed in cattle with PTB-associated multifocal lesions, SARS-CoV-2 disrupts essential cellular processes in human cells, including mRNA splicing and protein translation, and induces and regulates the innate immune response (58). Mitochondrial respiratory genes, cAMP-signaling components, and lipid-droplet regulators involved in thermogenesis are also required in cattle with multifocal lesions and this explains the genes sets overlapping thermogenesis (bta04714). Finally, several genes appeared DE in the transcriptome of cattle with multifocal lesions and associated with Parkinson disease (PD) (bta05012) because both conditions involve strong perturbations of mitochondrial respiration, proteasome function, and oxidative stress pathways even though one disease is infectious and the other neurodegenerative. MAP DNA has been detected by PCR in patients with PD, raising the possibility that chronic MAP infection may contribute to PD pathogenesis (59). One proposed link is the shared inhibition of autophagy (60). MAP is known to actively suppress autophagic pathways in infected macrophages to avoid intracellular degradation, while PD is characterized by impaired autophagy-lysosomal function leading to accumulation of misfolded α-synuclein and dysfunctional mitochondria.

## 5 Conclusions

This study provides the first transcriptomic comparison of PB from naturally MAP-infected cattle with multifocal lesions *vs* control cows. Upregulation of *OAS1Y, OAS2, ISG15, DHX58*, and *ZBP1* in PB suggests a coordinated innate defense network that detects intracellular bacteria and activates and regulates innate immune responses. Concurrent downregulation of ribosomal proteins and splicing factors suggests global translational and mRNA processing reprogramming. Enrichment of COVID-19 and Parkinson’s disease-related pathways suggest shared processes, highlighting common stress and defense mechanisms across diverse pathogens and pathological conditions. Overall, the study suggests that MAP persistence involves a complex balance of host innate immunity activation and regulation, translational reprogramming, and metabolic adaptation, offering novel insights into MAP pathogenesis and potential biomarkers.

## Data Availability

The datasets generated during the current study are available in the NCBI Gene Expression Omnibus (GEO) repository [https://www.ncbi.nlm.nih.gov/geo/] under the accession number GSE313938.
